# Trends and hotspots on the relationship between gut microbiota and Parkinson’s Disease: a bibliometric analysis

**DOI:** 10.3389/fcimb.2024.1421270

**Published:** 2024-09-30

**Authors:** Xuefeng Li, Xiaogang Hao, Chunhai Chen, Chao Zhai, Ting Pan, Xue Zhou, Yang Liu, Dalong Wu, Xinhua Chen

**Affiliations:** ^1^ Changchun University of Chinese Medicine, Changchun, China; ^2^ The Affiliated Hospital to Changchun University of Chinese Medicine, Changchun, China

**Keywords:** gut microbiota, Parkinson's disease, SCFAs, neuroinflammation, probiotics, bibliometric analysis

## Abstract

Parkinson’s disease (PD) is a neurodegenerative disorder that significantly impacts patients’ quality of life. Recent evidence has highlighted a complex relationship between the gut microbiota (GM) and PD. Understanding this relationship is crucial for potentially targeting GM in PD treatment and expanding therapeutic options. This study aimed to comprehensively analyze the global landscape, trends, and research focus on GM and PD using bibliometric analysis. Utilizing publications from the Web of Science Core Collection (WsSCC), bibliometric tools such as the R package ‘Bibliometrix,’ VOS viewer, and CiteSpace software were employed to assess parameters like yearly publications, countries/regions, institutions, and authors. Research trends and hotspots were identified through keyword analysis. The results revealed 1,161 articles published between 2013-2023, with China leading in publications (n=352, 30.31% of total), while the United States had a higher influence (H-index=58). The University of California System was the top institution in terms of publications (n=35), with the National Natural Science Foundation of China funding the most projects (n=172). Keshavarzian A and Sampson TR were the authors with the highest publication and co-citation counts, respectively. The *International Journal of Molecular Sciences* had the most articles published (n=48). Keyword analysis identified parkinson’s disease, gut microbiota, short-chain fatty acids, inflammation, and probiotics as main research topics. Biomarkers, ketogenic diet, and NF-κB were recent research hotspots and trends (2021-2023). The current study conducts an objective and comprehensive analysis of these publications, identifying trends and hotspots in the field of research. The findings offer valuable insights to scholars globally and in-vestigate potential therapeutic strategies for Parkinson’s Disease.

## 1 Introduction

Parkinson’s disease (PD) is the second most common neurodegenerative disorder, affecting an estimated 1%-4% of the global population over 60 years of age. (Tysnes and Storstein, 2017) Pathological changes in PD primarily involve abnormal aggregation of α-synuclein in dopaminergic neurons in the substantia nigra pars compacta (SNpc) and progressive loss of dopaminergic neurons. (Parkinson, 2002) PD is clinically characterized by motor symptoms, including resting tremor, rigidity, bradykinesia, and non-motor symptoms of gastrointestinal (GI) and urinary dysfunction, rapid eye movement(REM), constipation, depression, and cognitive dysfunction (Jankovic, 2008) The exact factors that trigger PD remain unknown; however, its development is influenced by a combination of genetic and environmental factors. While less than 10% of cases can be attributed to genetic factors, it is crucial to identify environmental risk factors for PD (Obeso et al., 2017; Marras et al., 2019).The gut serves as the interface through which environmental changes can impact the pathogenesis and progression of PD. Consequently, there is a growing body of research exploring the relationship between intestinal flora and PD, offering novel insights into PD research.

The gut microbiota, comprised of bacteria, viruses, fungi, and archaea, inhabits the human gastrointestinal tract (Falony et al., 2019). Research on the microbiome has rapidly advanced in the past two decades, thanks to improvements in DNA sequencing and microbiome bioinformatics. These advancements have revealed the various ways in which these microbial communities impact human health. The gut microbiome plays a crucial role in host physiology, influencing both healthy and diseased states, and can be seen as a key regulator of host pathology (Ding et al., 2018). Through the microbe-gut-brain axis (MGBA), the gut microbiota can affect the progression of PD (Forsyth et al., 2011; Hilton et al., 2014). Notably, individuals with PD have lower levels of anti-inflammatory bacteria like *Coprococcus*, *Roseburia*, and *Blautia spp* in their stool samples (Keshavarzian et al., 2015). Targeting gut microbes for disease treatment is a growing trend, with therapies such as probiotics (Leta et al., 2021), antibiotics (Mertsalmi et al., 2020), ketogenic diets (Jiang et al., 2023) and faecal microbial transplantation (FMT) (Zhao et al., 2021b) showing promise in PD animal models and clinical trials.

As research in GM and PD has advanced in recent years, the volume of scholarly literature in the field has grown significantly. A systematic and comprehensive literature review would be beneficial in gaining a deeper understanding of the current research landscape. With the increasing number of publications, bibliometric analyses can effectively summarize existing research and provide insights into the structure and quantitative aspects of a particular research area. These analyses can offer a broad overview of the current framework, highlighting key focuses and trends within the field (Zhang et al., 2024). Despite the growing body of literature, there is currently a lack of relevant bibliometric analyses on the correlation between GM and PD. Using bibliometric analyses, the study provides a comprehensive examination of annual outputs, collaborations, hotspots, research structure and emerging trends in the field aiming to offer valuable insights on the current status and future direction of PD and GM research.

## 2 Materials and Methods

### 2.1 Data Source and Search Strategy

**Figure 1:**
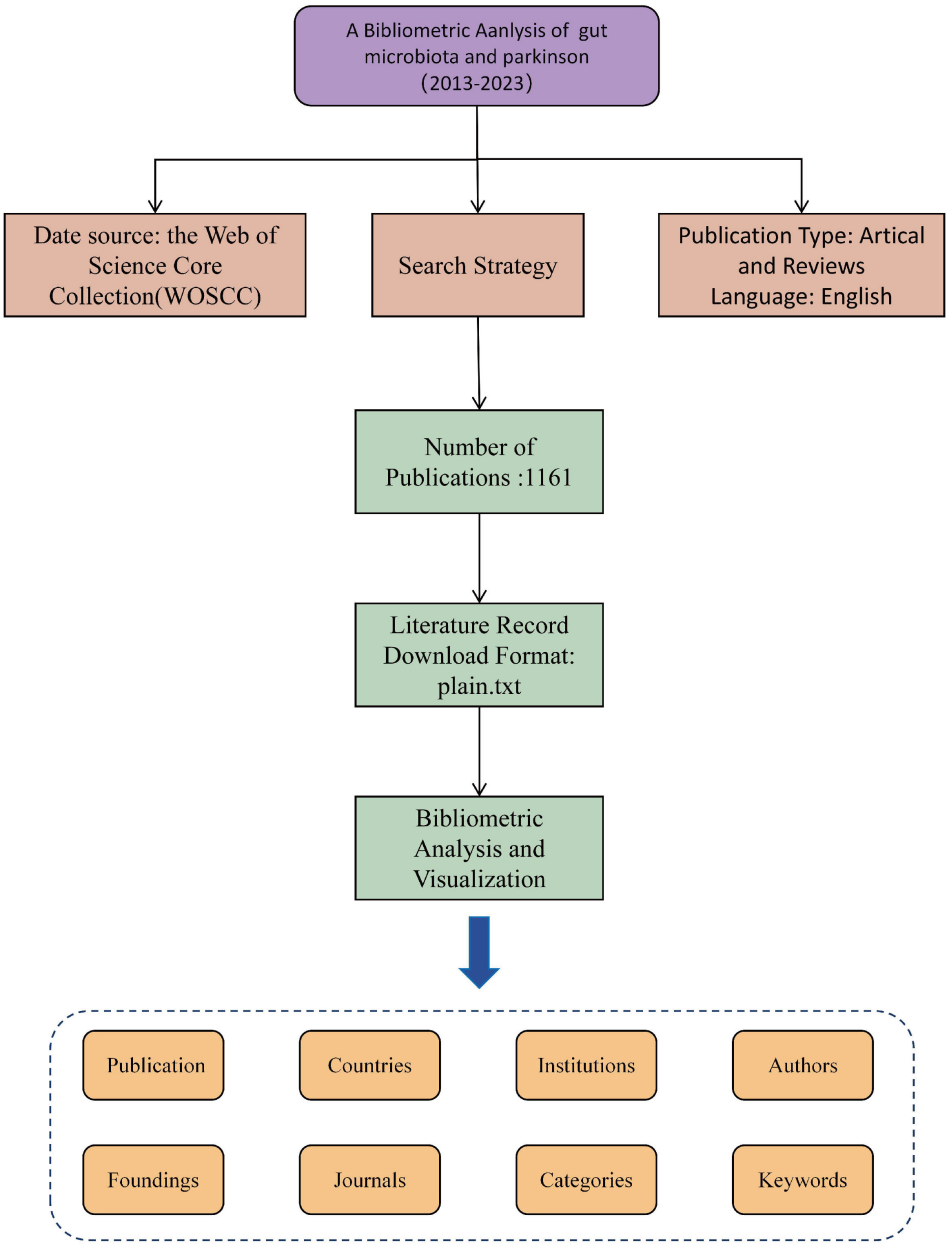
Flowchart of the selection process for the eligible literature.

Web of Science (WoS) is a widely utilized academic database with over 12,000 reputable journals and extensive citation records across various disciplines including natural sciences, biomedicine, engineering, and technology. (Chen, 2017; Birkle et al., 2020) The Web of Science Core Collection (WoSCC) serves as a high-quality literature database within WoS, offering up-to-date and reliable information. The publications referenced in this review were sourced from the WoSCC database on 15 February 2024, with all searches and data collection completed on the same day. The search strategy, detailed in **Supplementary File 1**, focused on publications up to 31 December 2023, primarily consisting of ‘primary research’ and ‘secondary review’ articals, including early access. Relevant publications were saved in plain.txt format for further analysis, encompassing complete records and cited references. (Pilkington, 2018) Following a comprehensive review, 1161 valid references were identified (**Figure 1**).

### 2.2 Software and related functions

In this study, the software tools CiteSpace (version 6.2.R4) and VOSViewer (version 1.6.16) were utilized for extracting and analyzing publication data, resulting in the creation of a knowledge graph. The software Orgin 2021 was employed to process the data and generate annual publication trends and annual citations graphs. CiteSpace, a citation analysis and visualization software, aids in visualizing the structure, distribution, and trends of information through scientific knowledge mapping (Synnestvedt et al., 2005). Within this review, CiteSpace was specifically used to produce visualizations of country, institution, author, journal, discipline, and keyword maps. Betweenness centrality quantifies the number of times a node acts as a bridge along the shortest path between two other nodes. Nodes with the highest centrality tend to be at the center of networks (Freeman, 1977).

VOSViewer, known for its user-friendly graphical representation and visually appealing images, offers various visual perspectives such as network, overlay, and density visualizations. (van Eck and Waltman, 2010) Pajek (version 5.17) is utilized for more advanced processing of scientific knowledge maps generated by VOSViewer. Additionally, Scimago can process data generated by VOSViewer, These software programs offer unique advantages and can effectively complement each other. The R package ‘bibliometrix’ (version 4.3.1) (https://www.bibliometrix.org) serves as an open-source software tool that provides a comprehensive set of functions for bibliometric analysis (Team, 2014). Within this review, Bibliometrix is utilized for keyword analysis and the generation of keyword clouds.

## 3 Results

### 3.1 The publication and citation trends

**Figure 2:**
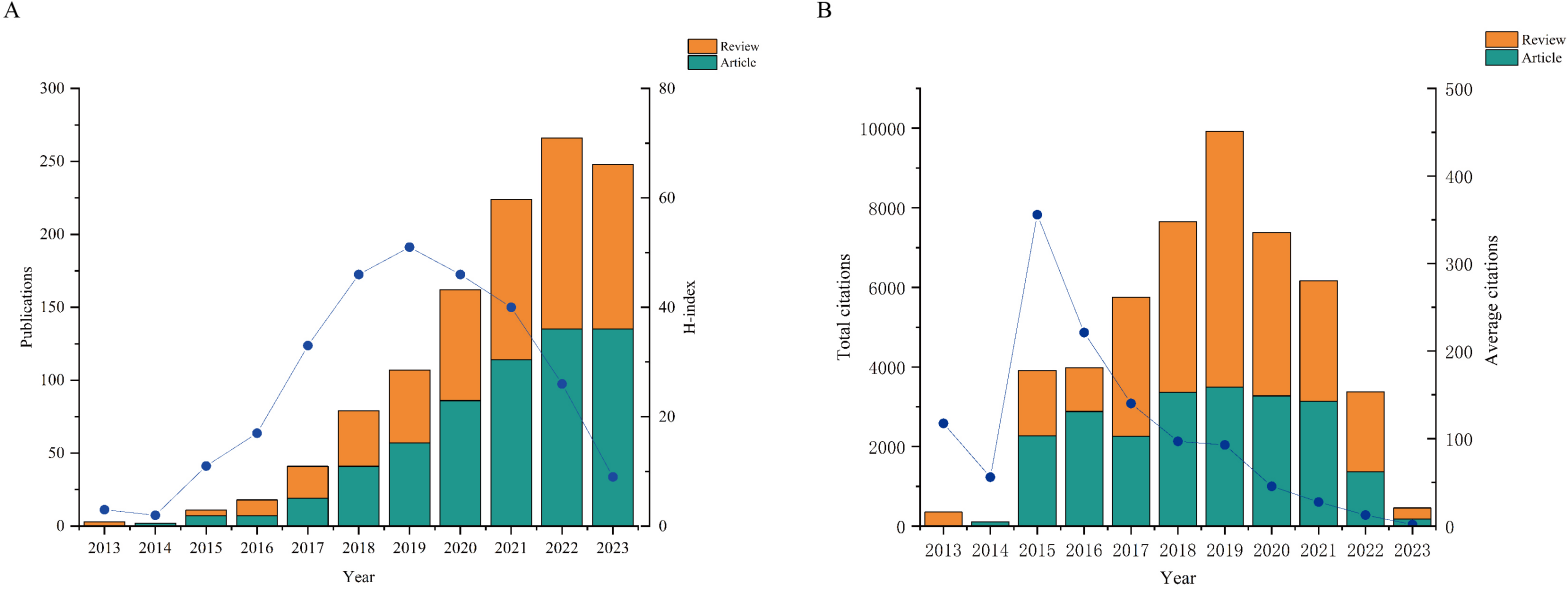
Quantities and trend of publications output and annual citations of GM in PD from 2013 to 2023. **(A)** Number of publications and H-index. **(B)** Annual citations and average citations.

In this study, a total of 1161 papers related to the involvement of gut microbes in PD were analyzed. Out of these, 603 were primary research and 558 were secondary review, accounting for 51.94% and 48.06%, respectively. The fluctuation in publication numbers over time reflects the field’s evolution. **Figure 2A** illustrates a consistent increase in the overall publication count annually, with a slight dip in 2023. The H-index serves as a measure of article impact, revealing the highest H-index in 2019. Correspondingly, the total number of citations for articles published in 2019 was also the highest, indicating their significant influence that year. Furthermore, the average number of citations peaked in 2015, suggesting a higher overall quality of articles from that year (**Figure 2B**).

### 3.2 Most active countries/areas

**Figure 3:**
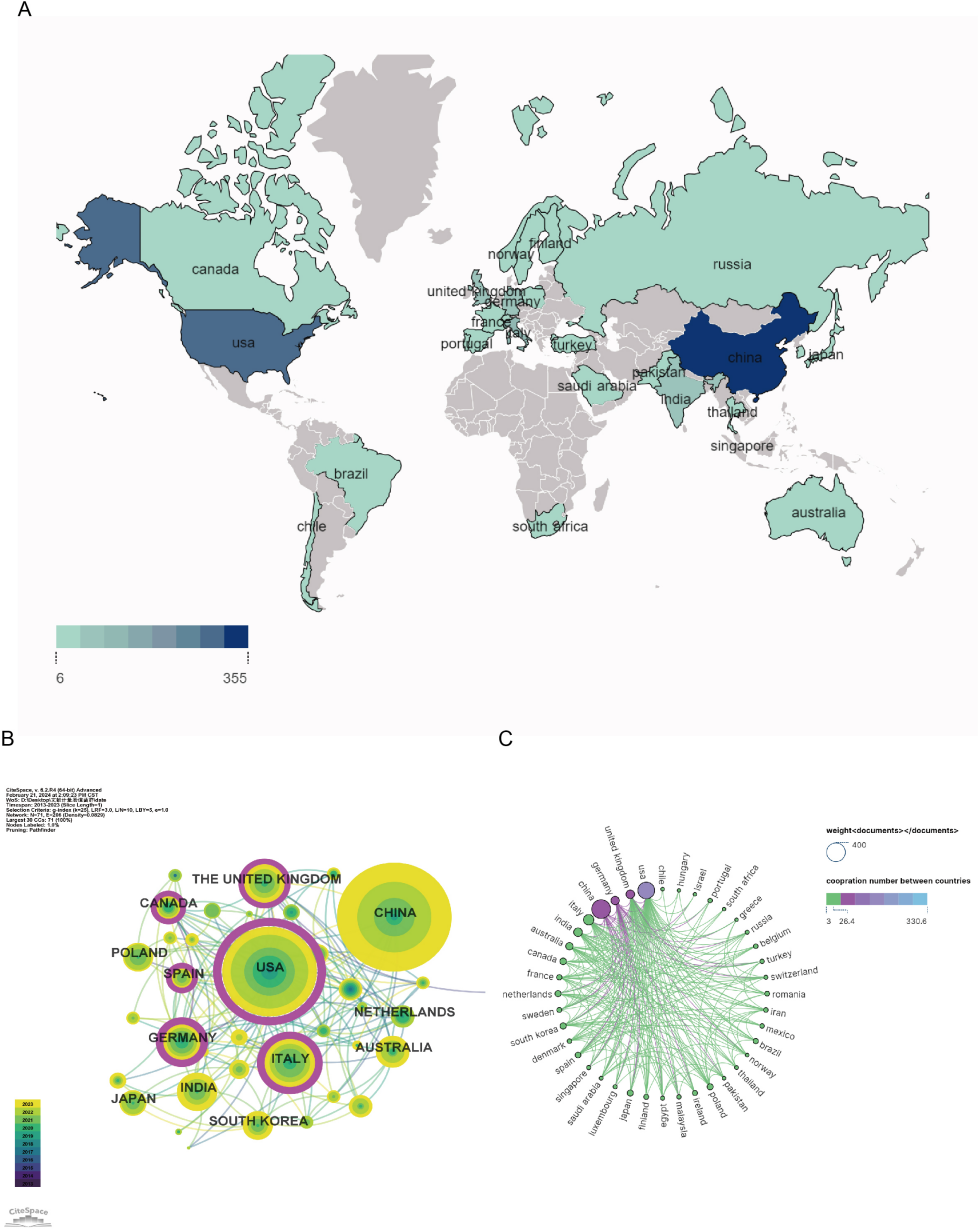
Geographical distribution and network of countries/regions in gut microbiota and PD. **(A)** Geographical map of publications; **(B)** Co-occurrence network of countries/regions; **(C)** Network diagram showing international cooperation.

A total of 73 countries/regions have contributed to research on GM and PD between 2013 and 2023. The top ten most active countries in terms of publications are listed in **Table 1**, with China leading with 352 publications (30.31% of the total), followed by the United States (278, 23.94%), Italy (102, 8.79%), the United Kingdom (67, 5.77%), and India (65, 5.6%). The geographical distribution of studies on GM and PD, depicted in **Figure 3A**, indicates a concentration in Europe, Asia, and North America. National and regional collaborative networks were analyzed using CiteSpace (**Figure 3B**), where nodes represent countries. Nodes with purple rings denote high centrality (≥ 0.1), signifying significant importance and influence. Countries like the United States, Italy, Germany, and the United Kingdom exhibit higher centrality. The US stands out with the most citations (n=15426) and highest h-index (n=58), reflecting its strong influence. Canadian publications have the highest average citations, indicating quality research output. Close collaborations are observed between the United States, the United Kingdom, China, and other nations, as illustrated in the country collaboration map in **Figure 3C**.

**Table 1:** Top 10 most countries in published research on GM and PD from 2013 to 2023.

Rank	Country	Documents	Citations	Average Article Citations	H-index
1	China	763	8019	24.45	44
2	USA	586	15426	55.09	58
3	Italy	402	5465	53.58	33
4	The United Kingdom	387	2473	40.54	25
5	India	386	1827	26.48	21
6	Germany	269	3372	53.52	24
7	Australia	198	1450	29.59	18
8	Canada	190	2385	62.76	21
9	South Korea	175	927	27.26	13
10	Poland	165	1299	40.59	17

### 3.3 Most active institutions and fundings

**Figure 4:**
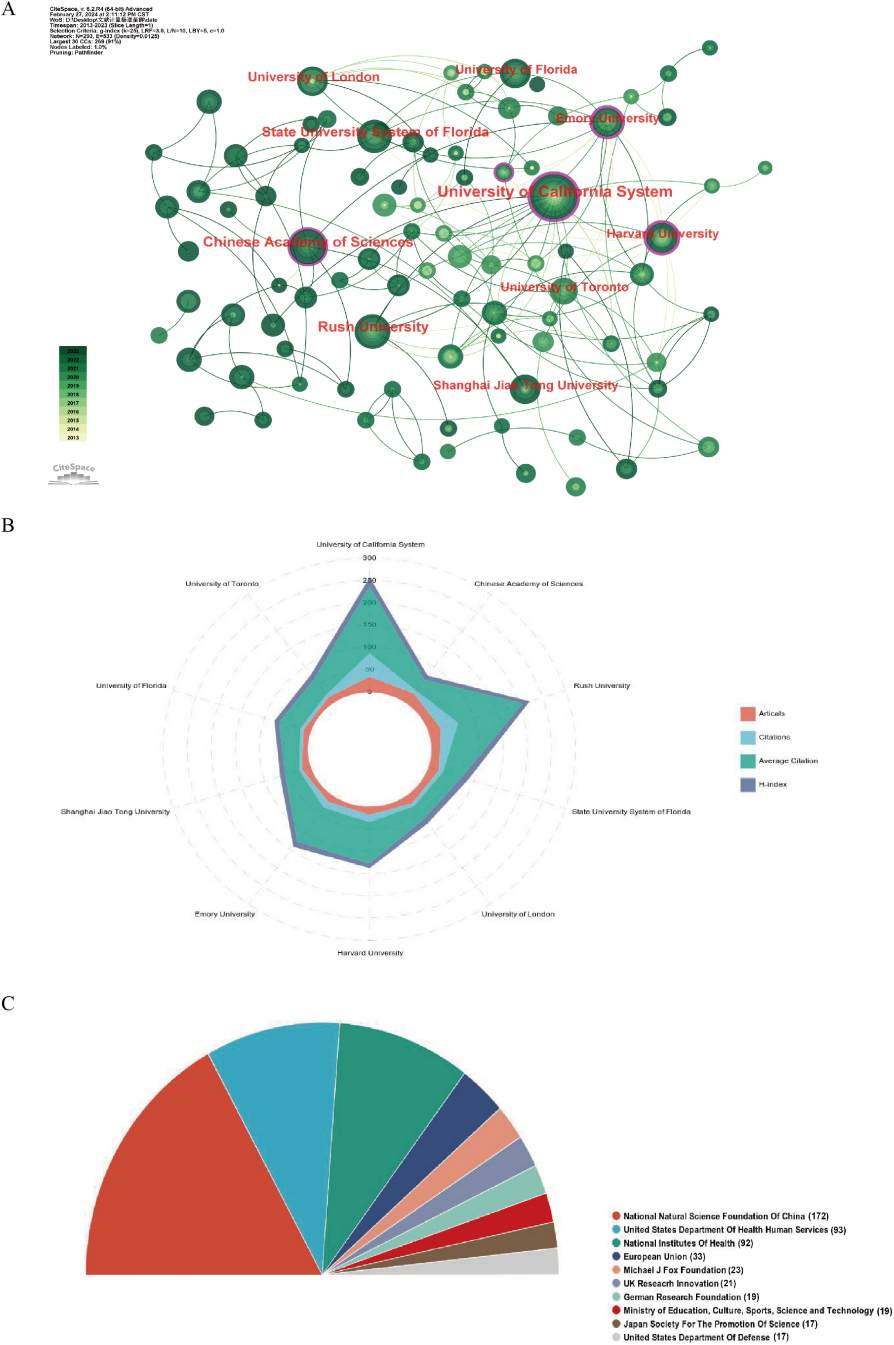
Visualization of institutions and funding related to the research on in gut microbiota and PD. **(A)** Occurrence of institutions; **(B)** The radar map ranking the top 10 based on the number of published papers, total citations, average citations, and H-index. **(C)** The top 10 most active funding agencies involved in this field.

A total of 1690 organizations were included in the study on GM and PD (**Table 2**). The University of California System had the highest number of articles (n=35), followed by the Chinese Academy of Sciences (n=27), Rush University (n=27), State University System of Florida (n=23), and University of London (n=21). Among the top ten active organizations, six were from the US, two from China, and one each from the UK and Canada. The University of California System led in total citations (n=5239), average citations (n=49.69), and H-index (n=20). Organizational collaboration was analysed using CiteSpace, where each node represents an organization, with node size indicating the number of papers and lines showing collaborative links. Nodes with purple circles denote higher centrality (centrality > 0.1), highlighting institutions like the University of California System, Chinese Academy of Sciences, and Rush University as central players in the collaborative network (**Figure 4A, 4B**). Adequate financial support is crucial for scientific development. The top ten active funding agencies include the National Natural Science Foundation of China (n = 172), the United States Department of Health Human Services (n = 93), and the National Institutes of Health (n = 92). The US and China are the primary sources of funding for this research area (**Figure 4C**).

**Table 2:** Top 10 publication institution related to GM and PD from 2013-2023.

Rank	Institution	Country	Articles	Citations	Average Article Citations	H-index
1	University of California System	USA	35	5239	149.69	20
2	Chinese Academy of Sciences	China	27	814	30.15	11
3	Rush University	USA	27	3809	141.07	15
4	State University System of Florida	USA	23	934	40.16	15
5	University of London	UK	21	737	35.1	13
6	Harvard University	USA	18	1667	92.61	10
7	Emory University	USA	17	1597	93.94	14
8	Shanghai Jiao Tong University	China	19	590	31.05	11
9	University of Florida	USA	16	738	46.13	10
10	University of Toronto	Canada	17	727	42.76	12

### 3.4 Most active authors and co-cited authors

**Figure 5:**
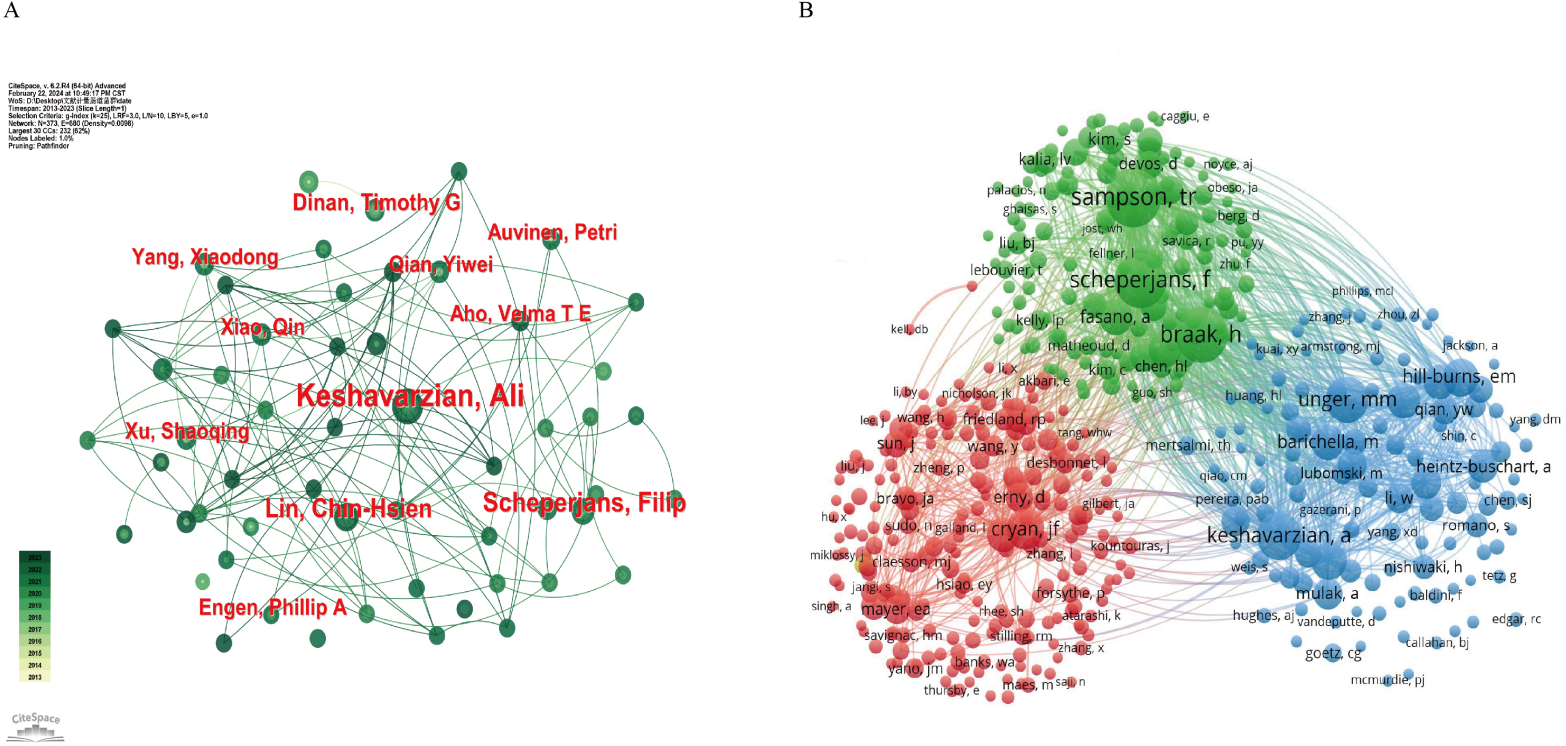
Network map of the correlation between GM and PD by author and co-cited author from 2013-2023. **(A)** Visualization map of main author cooperation network. **(B)** Network visualization map of co-cited authors in the correlation network.

**Figure 5** illustrates the author collaboration network, providing valuable insights for identifying potential research partners and prominent figures in the industry. As shown in **Table 3**, **Figure 5A**, Keshavarzian A stands out as the author with the highest number of publications (n=23), total citations (n=3638), and H-index (n=13) among the published articles. Dinan TG, on the other hand, has the highest average number of citations per article (n=447.25), indicating the high quality of his work. The co-citation analysis of authors (**Figure 5B**) reveals that out of 47458 co-cited authors, 5 authors have been cited more than 400 times (**Table 3**). Sampson TR leads the list with the highest number of co-citations (n=684), followed by Braak H (n=625), Scheperjans F (n=620), Keshavarzian A (n=458), and Unger MM (n=434). To visualize the co-citation network, authors with a minimum of 30 co-citations were included, with node size representing the number of co-citations received.

**Table 3:** Top 10 authors and co-cited authors related to GM and PD from 2013-2023.

Rank	Authors	Count	Total Citations	Average Citations	H-index	Co-Cited Authors	Citations
1	Keshavarzian A	23	3638	158.17	13	Sampson TR	684
2	Engen Phillip A	12	1188	99	9	Braak H	625
3	Yang XD	11	459	41.73	8	Scheperjans F	620
4	AhoV TE	11	1784	162.18	8	Keshavarzian A	458
5	Qin X	11	459	41.73	8	Unger MM	434
6	Qian YW	11	460	41.82	8	Cryan JF	348
7	Xu SQ	11	460	41.82	8	Hill-burns EM	306
8	Scheperjans F	11	645	58.64	9	Tan AH	303
9	Lin CH	9	492	54.67	7	Sun MF	271
10	Dinan TG	8	3578	447.25	8	Barichella M	270

### 3.5 Distribution of journals and categories

Journals are essential for academic evaluation, serving as a key platform for scholarly work. They offer valuable insights for sharing research findings within a specific field. In our study, we identified 430 journals assessing research, with 61 of them having more than 5 publications. **Table 4** presents the top 10 journals based on publication numbers. The *International Journal of Molecular Sciences* had the highest number of publications (n=48), followed by *Frontiers in Aging Neuroscience* (n=31) and *Journal of Parkinson’s Disease* (n=31). However, *Movement Disorders* stood out with the highest total citations (n=4171), mean number of citations (n=160.42), and H-index (n=8.6), indicating its significant impact and quality publications in the field of GM and PD. *NPJ Parkinson’s Disease* had the highest Impact Factor (IF) at 8.7. Notably, half of the top ten journals in the Journal Citation Reports (JCR) are classified as Q1.

**Table 4:** Top 10 journals published articles on GM and PD from 2013-2023.

Rank	Journal	Publications	Total citations	Average citations	H-index	IF	JCR
1	International Journal of Molecular Sciences	48	1274	26.54	16	5.6	Q2
2	Frontiers in Aging Neuroscience	31	578	18.65	12	4.8	Q2
3	Journal of Parkinsons Disease	31	788	25.42	17	5.2	Q2
4	NPJ Parkinsons Disease	28	832	29.71	13	8.7	Q1
5	Nutrients	27	879	32.56	12	5.9	Q1
6	Frontiers in Neuroscience	26	596	22.92	13	4.3	Q2
7	Movement Disorders	26	4171	160.42	21	8.6	Q1
8	Frontiers in Neurology	23	856	37.22	11	3.4	Q2
9	Frontiers in Cellolar and Infection Microbiology	18	538	29.89	9	5.7	Q1
10	Frontiers in Immunology	18	699	38.83	11	7.3	Q1

**Figure 6:**
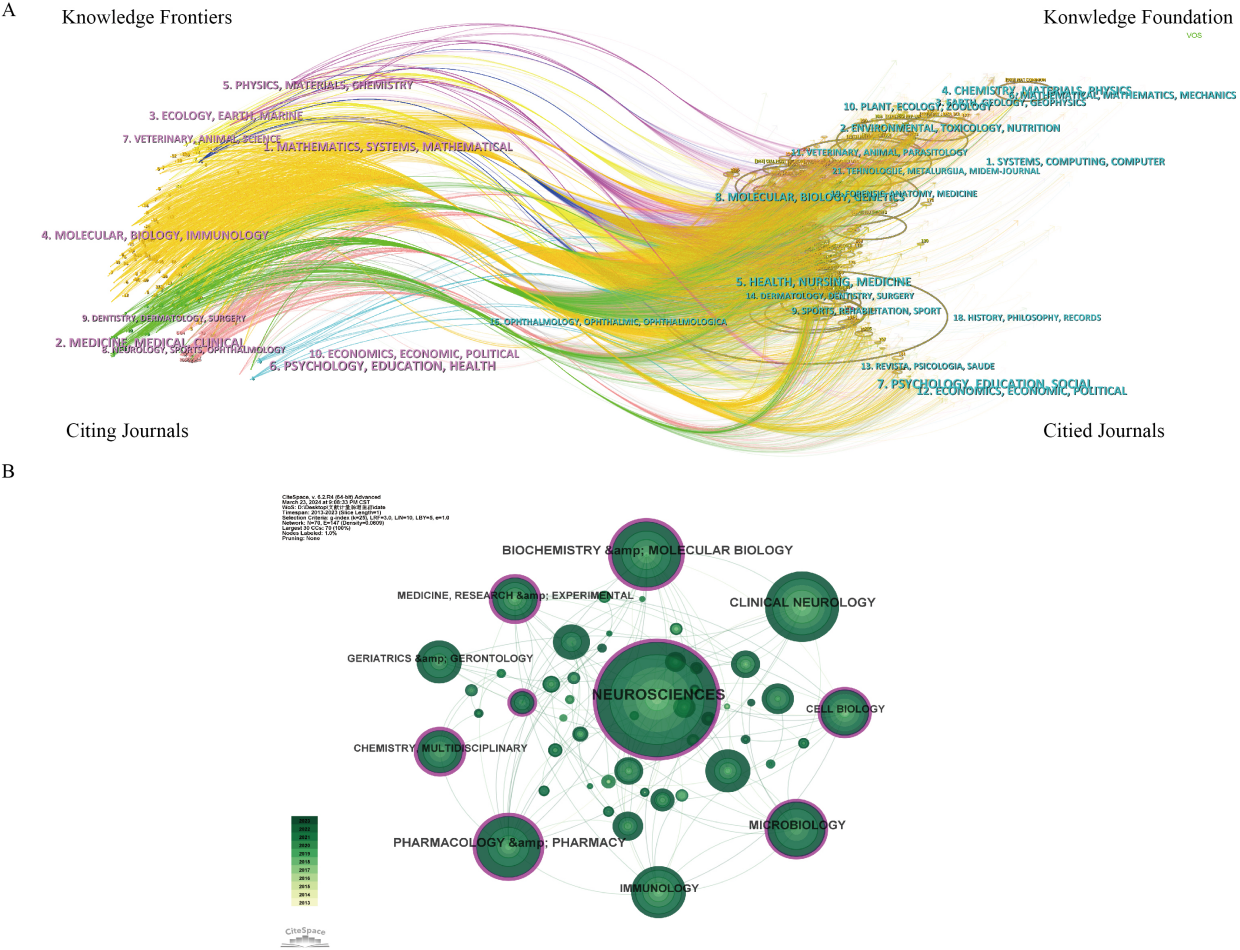
Journals and Categories visualization analysis related GM and PD from 2013 to 2023. **(A)** Dual-map overlay of journals. **(B)** Visualization map of categories network.

Dual-map overlay of journals in **Figure 6A** illustrates the distribution of journals where the cited literature is located on the left side, representing the main disciplines of scientific mapping. On the right side, it shows the distribution of journals corresponding to the cited literature, indicating which disciplines are primarily cited by scientific mapping. The findings reveal that the cited journals related to GM and PD are predominantly from fields such as molecular biology, immunology, neurology, sports, ophthalmology, pharmaceuticals, and clinical studies. These journals are frequently cited by publications in molecular biology, genetics, environment, toxicology, nutrition, psychology, education, and sociology. In general, GM and PD are closely linked to basic science, clinical medicine, nutrition, and social topics, suggesting the need for further multidisciplinary collaborations in the future. In **Figure 6B**, neurosciences emerge as the most represented research area (n=404 records, 34.8% of total articles), followed by Clinical Neurology (n=169, 14.56%), Biochemistry Molecular Biology (n=150, 12.92%), Pharmacology Pharmacy (n=132, 11.37%), and Microbiology (n=102, 8.8%).

### 3.6 Highly cited articles

The citation frequency of a paper is a crucial indicator of its high-impact status and extensive research interest. Highly cited literature, or the most frequently cited publications, receive significant scholarly attention (Chen, 2004). In **Table 5**, we present the top 10 most cited articles in the gut microbiota and PD field, highlighting their relevance and importance. Local citations are the number of publications cited in the local database, the higher the local citations, the more attention they receive from the field. Global citations are the total number of citations in the WOS database, the higher the global citations and the lower the local citations, the more important they are in the whole WOS database, but not in the discipline. These articles garnered local citations ranging from 165 to 556 and global citations ranging from 243 to 1995, with publication years mainly concentrated between 2015 and 2018. Among these articles, 8 focused on the specificity of the flora in PD patients, while only 2 centered on experimental studies in animal models. This emphasis on clinical studies in subsequent research influences the field’s direction and comprehension.

**Table 5:** Top 10 cited article on GM and PD from 2013-2023.

Rank	Title	Type	Year	First Author	Journals (IF-2023, JCR)	Local Citations	Global Citations
1	Gut Microbiota Regulate Motor Deficits and Neuroinflammation in a Model of Parkinson’s Disease	Artical	2016	Timothy R Sampson	Cell(64.5, Q1)	556	1995
2	Gut microbiota are related to Parkinson’s disease and clinical phenotype	Artical	2015	Filip Scheperjans	Movement Disorders(8.6, Q1)	531	1151
3	Colonic bacterial composition in Parkinson’s disease	Artical	2015	Ali Keshavarzian	Movement Disorders(8.6, Q1)	427	767
4	Short chain fatty acids and gut microbiota differ between patients with Parkinson’s disease and age-matched controls	Artical	2016	Marcus M Unger	Parkinsonism & Related Disorders(4.1, Q2)	397	690
5	Parkinson’s disease and Parkinson’s disease medications have distinct signatures of the gut microbiome	Artical	2017	Erin M Hill-Burns	Movement Disorders(8.6, Q1)	296	538
6	Functional implications of microbial and viral gut metagenome changes in early stage L-DOPA-naïve Parkinson’s disease patients	Artical	2017	J R Bedarf	Genome Medicine(12.3, Q1)	212	363
7	The nasal and gut microbiome in Parkinson’s disease and idiopathic rapid eye movement sleep behavior disorder	Artical	2018	Anna Heintz-Buschart	Movement Disorders(8.6, Q1)	209	330
8	Analysis of Gut Microbiota in Patients with Parkinson’s Disease	Artical	2017	V A Petrov	Bulletin of experimental biology and medicne(0.7, Q4)	178	301
9	Neuroprotective effects of fecal microbiota transplantation on MPTP-induced Parkinson’s disease mice: Gut microbiota, glial reaction and TLR4/TNF-α signaling pathway	Artical	2018	Meng-Fei Sun	Brain, behavior, and immunity(15.1, Q1)	170	373
10	Structural changes of gut microbiota in Parkinson’s disease and its correlation with clinical features	Artical	2017	Wei Li	Science China Life sciences(9.1, Q1)	165	243

### 3.7 Analysis of keywords

**Figure 7:**
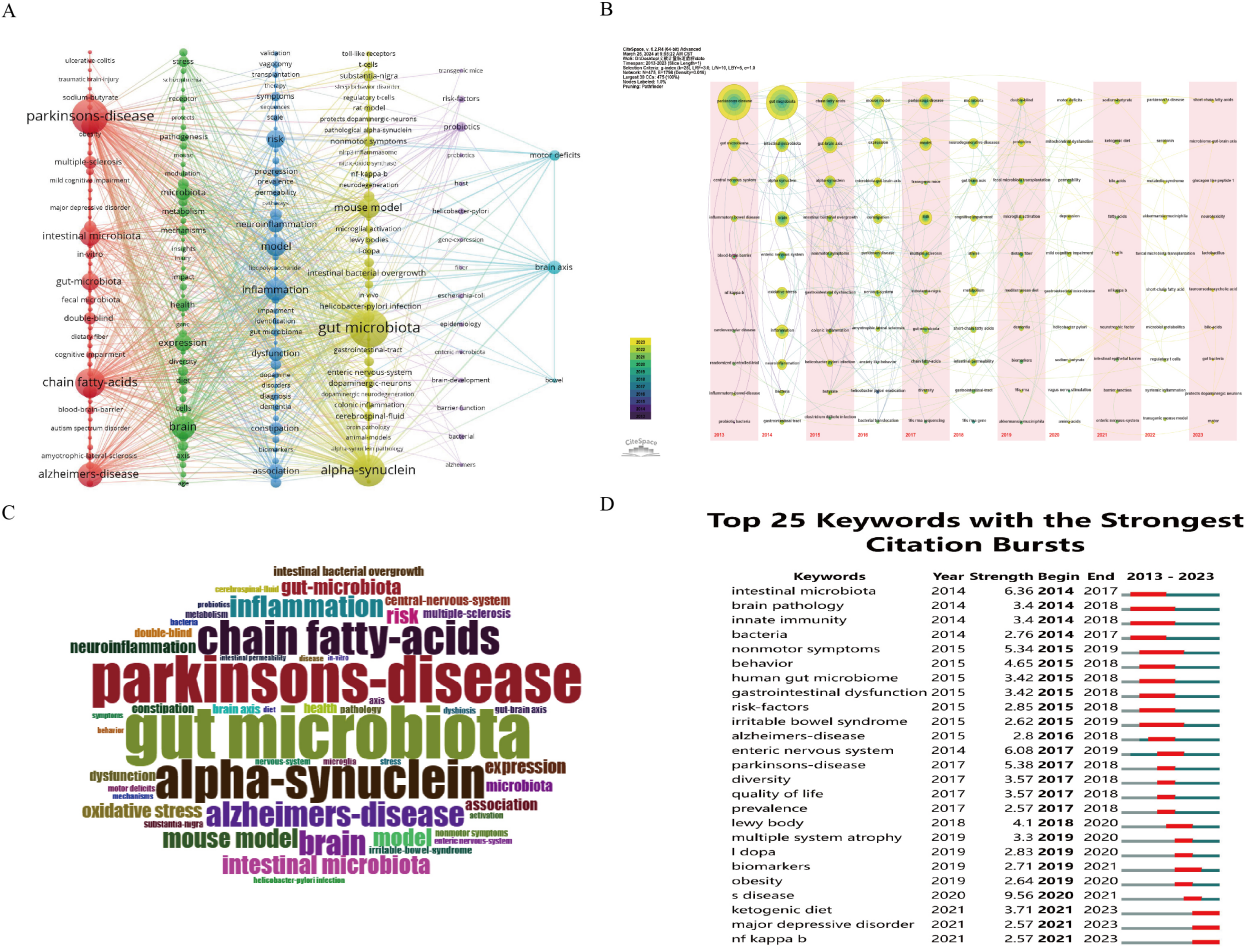
Visualisation of keywords related to GM and PDresearch from 2013-2023. **(A)** Network map of the frequency keywords(Top 223); **(B)** Time-based visualization of keyword variation in the field. Dots represented keywords, with larger dots indicated higher occurrence frequency of keywords, the clusters were labeled using different colors, and the links represented the co-occurrence of keywords; **(C)** Distribution of the top 50 author keywords; **(D)** The top 25 keywords with the strongest citation bursts. A blue bar represents the time period in which the keyword appeared; a red bar represents the interval in which the keyword was found to burst, indicating the start year, the end year and the duration of the burst.

A total of 4311 keywords were extracted from 1161 articles. Among these, only 233 keywords appeared more than 10 times, while the majority (69.29%) appeared only once, indicating a skewed distribution in keyword frequency. Gut microbiota was the most frequently occurring keyword (n=531), while PD had the highest link strength (Total Link Strength, TLS=3747) among all keywords. Cluster analysis of the keyword network graph revealed six distinct clusters (**Figure 7A**). The red cluster comprised 67 keywords, with parkinson’s disease, short-chain fatty acids (SCFAs), alzheimer’s disease, and intestinal microbiota appearing frequently, suggesting a significant role of SCFAs in intestinal and neurological diseases. The green cluster, consisting of 40 keywords, may be linked to PD complications and microbial metabolites, with nodes related to PD such as brain, expression, and stress, and gut microbial-related nodes like metabolism, diet, and diversity. The cyan cluster, with 42 keywords, highlighted Inflammation as a prominent node, indicating its crucial role in PD occurrence and development. The yellow cluster, containing 58 keywords, emphasized gut microbiota, alpha-synuclein, and mouse model as key nodes. Alpha-synuclein is extensively studied as a PD pathological product, while research using mouse models as PD models is a research hotspot. The purple cluster, with 15 keywords, focused on probiotics as a prominent node, with studies exploring compositional changes of bacteria and barrier function in the context of GM and PD relationship. Meanwhile, the blue cluster highlights the keywords intestines, brain axis, and motor deficits, indicating that intestinal microorganisms may impact motor deficits in PD through the gut-brain axis. The keyword time line visualization, as depicted in **Figure 7B**, is able to group nodes (keywords) within the same time zone and occurring at similar times. This visualization method illustrates how research hotspots evolve over time and can pinpoint the emergence of new hotspots. Specifically, it allows for a clear depiction of the development of research related to PD and intestinal flora. Keywords play a crucial role in highlighting hotspots within a research area, as demonstrated in **Figure 7C** where Parkinson’s Disease, gut microbiota, alpha-synuclein, and chain fatty acids are identified as the most commonly used keywords and focal points of research. In **Figure 7D**, identifying references with the strongest citation bursts can aid researchers in recognizing popular topics and shifts in research trends. The keywords with the most significant citation bursts were analyzed, revealing that intestinal microbiota, enteric nervous system, and Parkinson’s Disease exhibited the highest strength of citation bursts in recent years. Additionally, biomarkers, ketogenic, and NF kappa B were identified as the current hot research topics and trends over the past two years.

## 4 Discussion

Within the field of neurological disorders, there is a growing interest in the correlation between GM and PD. This study represents the first bibliometric analysis and visualization of the relationship between GM and PD.

### 4.1 Overview of development in the field of GM and PD

Over the past eleven years, there has been a significant increase in research focusing on the connection between GM and PD, demonstrating the ongoing progress in biomedical research. Our study offers a comprehensive overview of this field and highlights key contributors in terms of countries, institutions, authors, and journals. We analyzed 603 original research articles and 558 reviews related to the impact of GM and PD from 2013 to 2023 in the WOSCC. The number of publications has shown a consistent upward trend over the years, reaching a peak in 2022 and slightly declining in 2023. The H-index and total citations of these publications reached their highest point in 2019, a year when reviews had a greater impact. Conversely, only 13 articles were published in 2015, yet they garnered the highest average number of citations per publication (n=307), indicating a notable level of impact. This could be attributed to advancements in DNA sequencing and microbiome bioinformatics, which enabled more cost-effective and sophisticated analyses of the structural and functional microbiota, facilitating deeper and more convenient research on the complex multi-species system of gut microbes.

### 4.2 Analysis of countries, fundings, institutions, journals and authors

In the analysis of countries and regions, the number of publications by country is a reflection of their contribution to the research area. China and the United States stand out with deep academic accumulations and significant influence in this field. China has the highest number of publications (n=328) but a lower number of citations to papers (n=8019) compared to the United States (n=15426), possibly due to varying levels of financial and institutional support. Among the top ten funding sources, three are from the the United States, indicating a larger investment in PD and GM (**Figure 4C**). The substantial production of Chinese papers is supported by National Natural Science Foundation of China(NSFC). The the United States demonstrates the highest centrality (n=0.53) and close collaboration with other countries (**Figure 3C**). National economic backing and international partnerships are expected to further enhance the overall progress in this field.

The University of California System is recognized as the most productive institution globally, leading in publications, citations, and H-index. Research papers from this institution have explored the characterization of the gut microbiome in PD patients, investigating the impact of GM on levodopa metabolism, as well as the role of gut microbes in influencing motor deficits and neuroinflammation in PD models (Sampson et al., 2016; Hill-Burns et al., 2017; Maini Rekdal et al., 2019). When analyzing organizational collaborations, the United States institutions demonstrated a stronger partnership with global organizations, while Chinese organizations tended to collaborate more with domestic partners and less with international counterparts. Strengthening communication with international organizations could potentially enhance the production of high-quality research articles.

As for journal impact, the impact factor (Zimmerman et al., 2022) and JCR (Atallah et al., 2020) were potent indicators to value the journals’ impact. JCR Q1 and Q2 journals each accounted for half of the top 10 journals, with only three of them publishing more than 30 articles in GM and PD research. The *International Journal of Molecular Sciences* had the most publications. Despite being in the seventh position with 26 publications, *Movement Disorders*, a core journal in the field, had a much higher number of citations (n=4171) compared to other journals, indicating that the paper attracted the attention of a large range of authors and informed subsequent research.

For the GM and PD distinguished investigators, the top 10 active authors have each published a minimum of 8 articles. Among them, Ali Keshavarzian from the Department of Gastroenterology at Rush University Medical Centre, USA, stands out as a major contributor to the field, with significantly higher numbers of publications, total citations, and H-index compared to other authors. His article ‘Colonic Bacterial Composition in Parkinson’s Disease’ in *Movement Disorders* received a notable number of citations (n=767) and ranked as the third most highly cited. (Keshavarzian et al., 2015) The study concluded that both colon and faecal microbial communities in PD patients significantly differed from those in controls. The findings also suggested the presence of pro-inflammatory ecological dysregulation in PD patients, potentially triggering inflammation-inducing α-syn misfolding and contributing to the pathological progression of PD, thus reinforcing the correlation between GM and PD. As the research continues, Ali Keshavarzian published ‘Role of TLR4 in the gut-brain axis in Parkinson’s disease: a translational study from men to mice’ in the Gut in 2019 has only 298 citations. However, it demonstrated that TLR4-mediated inflammation plays an important role in gut and brain inflammation, which may be one of the key factors leading to neurodegenerative lesions in PD. It breaks new directions for exploring the role of the brain-gut axis in PD (Perez-Pardo et al., 2019).Then, Ali Keshavarzian published ‘An open label, non-randomized study assessing a prebiotic fiber intervention in a small cohort of Parkinson’s disease participants’ in Nature Communications in 2023. This proof-of-concept study provides the scientific rationale for future studies evaluating the potential of a microbiota-directed prebiotic intervention as a disease-modifying therapeutic approach in PD patients (Hall et al., 2023). Co-citation analysis of top authors with at least 270 co-citations revealed notable contributions to the field. Sampson Timothy R (684 co-citations) emerged as the most cited author, followed by Braak Heiko (625 co-citations) and Filip Scheperjans (620 co-citations). Sampson Timothy R’s research highlighted the influence of gut microbiota on brain development, function, and behavioral control through various pathways connecting the gut to the central nervous system. (Sampson and Mazmanian, 2015) Additionally, a study involving Thy1-αSyn (ASO) mice showed that mice receiving faecal transplants from PD patients exhibited more severe motor deficits compared to those transplanted with healthy human faeces, indicating that alterations in the human microbiome could be a risk factor for PD (Sampson et al., 2016). With ongoing studies, Sampson Timothy R thought red blood cell-derived vesicles may also be a mediator of bi-directional α-Syn propagation between peripheral sites and the CNS or even an origin of a-syn corruption, expanding the foundation for the continued work in PD etiopathology in peripheral systems (Sampson, 2024).

### 4.3 Research hotspots and trends

Gut microbiota and PD have garnered significant attention from researchers globally. Through an analysis of the top ten most cited articles, as well as keyword co-occurrence, clustering, and emerging trends, it was revealed that current research focuses on key areas such as the microbe-gut-brain axis, short-chain fatty acids (SCFAs), probiotics, and inflammation (**Figure 7**). Increasingly, studies are shedding light on the significant impact of gut microbes on neurodevelopment and the central nervous system (Sharon et al., 2016). In the case of PD, the second most prevalent neurodegenerative disease, numerous clinical studies (Aho et al., 2019; Wallen et al., 2022; Huang et al., 2023) and experimental studies (Sampson et al., 2016; Wang et al., 2021; Zhao et al., 2021b) have demonstrated the influence of gut flora on the onset and progression of PD. As a result unraveling these mechanisms is crucial for the development of effective treatment and prevention strategies for PD influenced by gut microbiota.

#### 4.3.1 Microbiota-gut-brain axis

Disruption of the gut microbiota balance has been linked to various human diseases, such as gastrointestinal, neurological, respiratory, and cardiovascular diseases (Lynch and Pedersen, 2016). Gut microbiota play a crucial role in the gut-brain connection and are often referred to as the ‘second brain’ of the human body (Morais et al., 2021). The enteric nervous system (ENS) communicates with the central nervous system (CNS) through sensory and motor neurons, as well as neurotransmitters, primarily relying on vagal afferent and efferent fibers for neural pathways (Forsythe et al., 2014). The gut microbiota produces neurotransmitters like acetylcholine, serotonin, and dopamine, as well as neuroactive molecules such as SCFAs, which can transmit signals to the brain via the endocrine pathway (Carabotti et al., 2015; Dinan et al., 2015). Scientists have pointed out a strict relationship between the brain and the gut microbiota, known as the microbiota-gut-brain (MGB) axis. Indeed, MGB axis alterations are known to affect both intestinal epithelial barrier (IEB) and blood-brain barrier (BBB) integrity, triggering an immune reaction, and potentially increasing brain inflammation (Parashar and Udayabanu, 2017; Bellini et al., 2023). PD is affected by MGB as a neuroinflammatory disease, with dysregulation of gut microbial ecology serving as a significant risk factor and determinant of PD (Santos et al., 2019). Recent clinical research has demonstrated associations between gut microbiota and PD characteristics, including onset time (Lin et al., 2018), disease duration (Keshavarzian et al., 2015), disease stage (Pietrucci et al., 2019), motor symptoms (Hegelmaier et al., 2020) and non-motor symptoms (Heintz-Buschart et al., 2018). Specifically, for motor symptoms in PD, the abundance of *Lactobacillus* has been correlated with the degree of impaired motor function (Barichella et al., 2019), while the abundance of the *Enterobacteriaceae* family has been linked to walking difficulties and akinetic-rigid subscores (Aho et al., 2019). Exploring the mechanisms through which the microbiota-gut-brain axis influences the nervous system can enhance our understanding of PD etiology, making it a burgeoning research area in recent years.

#### 4.3.2 SCFAs

Short-chain fatty acids, such as formate, acetate, propionate, and butyrate, are significant products of gut microorganisms. These compounds play a crucial role in enhancing intestinal health through various mechanisms, including the maintenance of intestinal barrier integrity, mucus production, and possessing antioxidant and anti-inflammatory properties. (Dinan et al., 2015) Research has shown that levels of acetate, propionate, and butyrate are notably reduced in fecal samples from individuals with PD (Baert et al., 2021). Studies on animal models suggest that butyrate may potentially mitigate neurological damage in PD by inhibiting the over-activation of nigrostriatal microglia and suppressing the expression of inflammatory factors like TNF-α, IL-6, and IL-1β (Hou et al., 2021). Additionally, sodium butyrate has been found to increase dopamine levels and alleviate dyskinesia in a drosophila model of PD induced by rotenone. In a mouse model of PD induced by MPTP, long-term administration of phenylbutyrate has shown to increase brain DJ-1 activity, reduce α-syn aggregation, and prevent motor degeneration and cognitive deficits (Zhou et al., 2011). However, the role of short-chain fatty acids in PD is still a topic of debate. Recent studies have indicated that plasma levels of SCFAs remain unchanged or even elevated in PD patients (Shin et al., 2020), despite decreased fecal levels. This discrepancy may be attributed to intestinal barrier dysfunction, allowing SCFAs to enter the systemic circulation (Yang et al., 2022). In animal models, supplementation of SCFAs to germ-free mice has been shown to exacerbate α-synuclein-mediated neuroinflammation and motor deficits (Sampson et al., 2016). Sodium butyrate has been found to enhance nigrostriatal microglia and astrocyte activation in PD mice, further intensifying neuroinflammation and leading to the development of motor deficits and dopaminergic neuron loss in PD mice (Qiao et al., 2020). The exact causal relationship between SCFAs and PD is yet to be fully understood, necessitating further research to elucidate the specific modulatory effects of SCFAs on PD.

#### 4.3.3 Probiotics

The investigation of probiotics in GM and PD has garnered significant attention in recent research. Probiotics, which are primarily comprised of naturally occurring bacteria in the gut, such as *Lactobacillus*, *Bifidobacterium*, and *Saccharomyces*, have shown promise in reversing microbiological changes in the gut associated with PD (Sanders et al., 2019).This restoration of gut function can help inhibit leaky gut and neuroinflammation. Certain bacterial strains like *Bifidobacterium* and *Lactobacillus* have demonstrated the ability to reduce neuroinflammation and abnormal aggregation of α-synuclein through the production of vitamins, antioxidants, and bioactive molecules, ultimately decreasing oxidative stress (LeBlanc et al., 2013). *Bifidobacterium breve* Bif11can regulate SCFA levels and intestinal epithelial permeability, increased the tyrosine hydroxylase levels, reduced pro-inflammatory markers and decreased oxidative and nitrosative stress in the mid brain of MPTP-lesioned rats (Valvaikar et al., 2024). Clinical meta-analyses have shown that the potential value of probiotics in improving constipation symptoms in PD patients, encouraging probiotics to be utilized alone or in combination with other therapies in clinical practice for PD patients (Jin et al., 2024). Another study showed decreased bloating and abdominal pain in PD patients when supplemented with *Lactobacillus acidophilus* and *Bifidobacterium* infantis for 3 months in tablet form (Georgescu et al., 2016). Clinical studies have indicated that a combination of probiotics including *Lactobacillus acidophilus*, *Bifidobacterium*, and *Lactobacillus fermentum* in tablet form can lead to improvements in PD symptoms, as evidenced by reduced MDS-UPDRS scores and levels of inflammatory markers like high-sensitivity C-reactive protein and malondialdehyde, while increasing glutathione levels (Tamtaji et al., 2019). Furthermore, co-administration of two anti-inflammatory agents, probiotics and vitamin D, may reduce disease severity and complications in patients with PD, making it a promising and potentially effective treatment option (Zali et al., 2024). Despite the promising outcomes from different preclinical and clinical studies, several issues and concerns with probiotic supplementation still need to be addressed (Fasano et al., 2015; Maini Rekdal et al., 2019). The major current problems include:1) the risk of developing fungaemia or bacteraemia, 2) the need of a personalized assessment when prescribing probiotics, and 3) the possible interactions with levodopa in the small intestine, which in turn reduces the amount of drug reaching the brain. Hence, to address these uncertainties, rigorous clinical trials are essential to determine the optimal combination of probiotic strains, dosages, and treatment durations in order to evaluate the efficacy and safety of long-term supplementation.

#### 4.3.4 Inflammation

Inflammation is a prominent area of research in studies on GM and PD. Two key indicators of PD are progressive dopaminergic neurodegeneration and significant neuroinflammation in the substantia nigra striata pathway (Bloem et al., 2021). Dysbiosis of the intestinal microflora can result in decreased expression of intestinal tight junction proteins, leading to a ‘leaky gut’ phenomenon that allows inflammatory factors to enter the bloodstream (Sun and Shen, 2018). These pro-inflammatory factors can disrupt the blood-brain barrier (BBB), permitting inflammatory cytokines to reach the SN and contribute to neuroinflammation and the death of dopaminergic neurons (Elahy et al., 2015; Gray and Woulfe, 2015). Clinical investigations have shown reduced levels of anti-inflammatory genera *blautia* and *coprococcus* spp. in fecal samples from PD patients, indicating a shift towards a more inflammatory gut environment (Keshavarzian et al., 2015). Alterations in gut microbiota composition, including an increase in certain microorganisms like *Verrucomicrobia* and *Bacteroides*, have been associated with elevated levels of inflammatory markers such as level of TNF-α and IFN-γ in PD patients (Lin et al., 2019). As a result, the intrinsic neuroprotective mechanism in the gut may contain pathways that could also induce neuroprotection in the brain, when in contact with external pathogens, and, as many of the symptoms of PD and other neurode­generative diseases arise due to neuroinflammation, research on this intrinsic neuroprotective mechanism should be further stimulated (Claudino Dos Santos et al., 2023). Toll-like receptors (TLRs) play a crucial role in the interaction between GM and PD, with elevated levels of TLR2 and TLR4 observed in the blood and brain of PD patients (Drouin-Ouellet et al., 2015; Kouli et al., 2019). TLR2 activation can trigger a neuroinflammatory response and promote α-synuclein aggregation through the TLR2/MyD88/NF-kB pathway (Cheng et al., 2023), while TLR4 is involved in the clearance of α-synuclein and initiates microglial responses in PD (Fellner et al., 2013). Given the significance of TLR4 in PD, research has focused on targeting and modulating this pathway as a potential therapeutic approach for PD (Zhao et al., 2021a).

### 4.4 Strength and limitation

This bibliometric study systematically analyzes the basic situation, research hotspots, and trends of GM and PD interactions from a visualization perspective. The study aims to provide a comprehensive reference for researchers working on this topic. Various bibliometric software tools were utilized to explore different dimensions of research hotspots, leading to more accurate and objective findings. However, the study has some limitations. It only included original articles and reviews in English from the WoSCC, potentially missing publications not included in the database. Moreover, the study focused on original research and reviews in English, possibly limiting the number of publications retrieved and omitting some relevant works. The inability of the bibliometric software to differentiate between studies on human and animal models is another constraint. Additionally, the presence of duplicate names for some authors and the potential affiliation of authors with multiple universities as honorary or part-time faculty members pose challenges. Nevertheless, the study aims to provide an accurate depiction of the current research status and general trends in this field, offering a valuable foundation for neuroscientists and other researchers to identify research priorities and emerging trends in the relationship between GM and PD.

## 5 Conclusions

Based on reviews and original articles by WOSCC in the field, we have gained a comprehensive and systematic understanding of the correlation between GM and PD through bibliometrics. Research related to GM and PD has emerged as a prominent area of study, with publications on this topic steadily increasing over the past 11 years. While China leads in the number of publications, the United States holds the most influence in this field. Collaborative efforts among various institutions and authors have driven the dynamic growth of research in this area. The microbial-gut-brain axis and inflammation play key roles in elucidating how GM impacts PD. Therapeutic strategies targeting the gut microbiota, such as probiotics, SCFAs, ketogenic diets, and FMT, have demonstrated positive effects on PD. It is essential to continue conducting animal experiments and clinical trials to further evaluate the efficacy of these strategies.
