# Supplementary material for: Trends and hotspots on the relationship between gut microbiota and Parkinson’s Disease: a bibliometric analysis
Source: Front Cell Infect Microbiol. 2024 Sep 30;14:1421270. doi: 10.3389/fcimb.2024.1421270 (PMC11472709; doi:10.3389/fcimb.2024.1421270)
Supplement: Supplementary file 1 [file Table1.docx]

**Search Strategy**

**TS=(“Gastrointestinal Microbiome” OR “Gastrointestinal Microbiomes” OR”Microbiome, Gastrointestinal” OR”Gut Microbiome” OR “Gut Microbiomes” OR”Microbiome, Gut” OR “Gut Microflora” OR “Microflora, Gut” OR “Gut Microbiota” OR “Gut Microbiotas” OR “Microbiota, Gut” OR “Gastrointestinal Flora” OR “Flora, Gastrointestinal” OR “Gut Flora” OR “Flora, Gut” OR “Gastrointestinal Microbiota” OR “Gastrointestinal Microbiotas” OR “Microbiota, Gastrointestinal” OR “Gastrointestinal Microbial Community” OR “Gastrointestinal Microbial Communities” OR “Microbial Community, Gastrointestinal” OR “Gastrointestinal Microflora” OR “Microflora, Gastrointestinal” OR “Gastric Microbiome” OR “Gastric Microbiomes” OR “Microbiome, Gastric” OR “Intestinal Microbiome” OR “Intestinal Microbiomes” OR “Microbiome, Intestinal” OR “Intestinal Microbiota” OR “Intestinal Microbiotas” OR “Microbiota, Intestinal” OR “Intestinal Microflora” OR “Microflora, Intestinal” OR “Intestinal Flora” OR “Flora, Intestinal” OR “Enteric Bacteria” OR “Bacteria, Enteric”) AND TS=(“parkinson” OR “parkinson disease”)**
